# Morphological and Structural Analysis of Polyaniline and Poly(*o*-anisidine) Layers Generated in a DC Glow Discharge Plasma by Using an Oblique Angle Electrode Deposition Configuration

**DOI:** 10.3390/polym9120732

**Published:** 2017-12-20

**Authors:** Bogdan Butoi, Andreea Groza, Paul Dinca, Adriana Balan, Valentin Barna

**Affiliations:** 1 Faculty of Physics, University of Bucharest, 405 Atomistilor Street, 077125 Magurele, Romania; bogdan.butoi@g.unibuc.ro (B.B.); dincapaulpavel@yahoo.com (P.D.); 2 National Institute for Laser, Plasma and Radiation Physics, 409 Atomistilor Street, 077125 Magurele, Romania; 3 Nano-SAE Research Centre, Faculty of Physics, University of Bucharest, 077125 Magurele, Romania; andronie@3nanosae.org

**Keywords:** polyaniline layers, poly(*o*-anisidine) films, DC plasma polymerization method, conductivity measurements

## Abstract

This work is focused on the structural and morphological investigations of polyaniline and poly(*o*-anisidine) polymers generated in a direct current glow discharge plasma, in the vapors of the monomers, without a buffer gas, using an oblique angle-positioned substrate configuration. By atomic force microscopy and scanning electron microscopy we identified the formation of worm-like interlinked structures on the surface of the polyaniline layers, the layers being compact in the bulk. The poly(*o*-anisidine) layers are flat with no kind of structures on their surfaces. By Fourier transform infrared spectroscopy we identified the main IR bands characteristic of polyaniline and poly(*o*-anisidine), confirming that the polyaniline chemical structure is in the emeraldine form. The IR band from 1070 cm^−1^ was attributed to the emeraldine salt form of polyaniline as an indication of its doping with H^+^. The appearance of the IR band at 1155 cm^−1^ also indicates the conducting protonated of polyaniline. The X-ray diffraction revealed the formation of crystalline domains embedded in an amorphous matrix within the polyaniline layers. The interchain separation length of 3.59 Å is also an indicator of the conductive character of the polymers. The X-ray diffraction pattern of poly(*o*-anisidine) highlights the semi-crystalline nature of the layers. The electrical conductivities of polyaniline and poly(*o*-anisidine) layers and their dependence with temperature are also investigated.

## 1. Introduction

Polyaniline and poly(*o*-anisidine) are conducting polymers that present a well-known interest in different research areas due to their high potential applications in displays and molecular devices, smart windows, energy storage systems, and as corrosion protective layers [1].

The polyaniline can be synthetized in different chemical structural forms, such as leucoemeraldine, emeraldine base, pernigraniline or emeraldine salt that present specific electrical behaviors. Leucoemeraldine is electrically insulated with no double bonds between the aromatic rings and N–H groups. Emeraldine base has few N–H groups in its main chain being partially oxidized and insulated. A fully-oxidized compound is the pernigraniline having no N–H groups in its main chain structure and, thus, no conducting properties. Protonation of the emeraldine base to emeraldine salt induces an insulator to conductor conversion, the transition taking place on the –NH– groups [2,3]. The poly(*o*-anisidine) is a derivative form of aniline, having a methoxy (–OCH_3_) group substituted at the ortho-position on the benzene ring [4].

Over time, these conducting polymers have been produced by chemical, electrochemical, or plasma polymerization techniques [5,6,7]. Their synthesis represents an attractive topic as their structural properties, like morphology and chemical form, are influenced by the type of the polymerization method and the specific generation conditions.

By DC or RF plasma deposition methods, the polymerization of conducting monomers involves complex chemical reactions mainly due to electron impact dissociation and ionization of chemical species. In [1] it was showed that the polymerization of the organic compounds in glow discharges take place by a free radical mechanism, the ionization being small. Thus, the radicals produced in the plasma are combined and high molecular weight cross-linked polymers can be generated.

In this paper we present the results on the morphological and physicochemical characterization of the polyaniline (PANI) and poly(*o*-anisidine) (POA) layers generated in a DC glow discharge plasma. The electrode discharge configuration has the particularity that the substrate holder can be positioned at different oblique inclination angles to the anode. The vaporized aniline or *o*-anisidine monomers are injected into the plasma through the anode.

The influence of the deposition plasma conditions, such as the monomer injection temperature into the plasma, anode-substrate inclination angle, and discharge current intensity, on the physical and chemical properties of the polymer layers are analyzed. The morphology of the polymer layers was investigated by scanning electron microscopy (SEM) and atomic force microscopy (AFM). The structure and the chemical composition of the polymers were analyzed by Fourier transform infrared spectroscopy (FTIR) and X-ray diffraction (XRD). The dependence with temperature of the electrical conductivities of polyaniline and poly(*o*-anisidine) layers was also analyzed.

## 2. Materials and Methods

### 2.1. Materials

Aniline and *o*-anisidine monomers in liquid form (Sigma-Aldrich Chemistry, Dorset, UK) were used as precursors for the synthesis of polyaniline and poly(*o*-anisidine) polymeric layers in a DC glow discharge plasma using various deposition parameters.

### 2.2. Deposition Technique of Polyaniline and Poly(o-Anisidine) Layers

**Figure 1:**
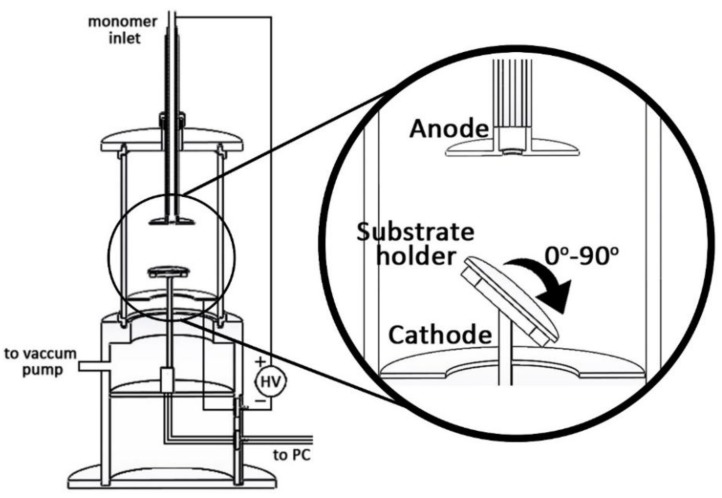
Experimental setup of the employed DC plasma reactor [6,7].

The layers of polyaniline (PANI) and poly(*o*-anisidine) (POA) were synthesized in the plasma of a DC glow discharge produced in the vapors of the monomers, with no buffer gas, using the experimental setup presented in Figure 1.

The DC plasma reactor consists of two ring-shaped electrodes, mounted in parallel, namely the anode, through which a vaporized liquid monomer is directly injected, and the cathode, through which the sample’s substrate holder is positioned, as shown in Figure 1. The liquid monomers (aniline and *o*-anisidine) were heated to 20 °C, and consecutively to 50 °C, and the vapors were injected (due to the pressure difference between the vacuum chamber and the monomer container) through a pipe from the top of the reactor centrally positioned to the anode. There is no buffer gas in the vacuum chamber, the pressure being about 5 × 10^−2^ torr.

The sample substrates were placed on the holder at 5 and 10 cm from the anode. The position of the samples holder relatively to the anode and its angle of inclination (0°, 45°, and 90°) are controlled precisely through a six servo-mechanism system.

In these experimental conditions, applying a voltage between the anode and the cathode, a glow discharge plasma in the aniline or *o*-anisidine vapors is produced.

The experimental conditions used for the plasma deposition of polyaniline (PANI) and poly(*o*-anisidine) (POA) layers on Si substrates are presented in Table 1.

The polymerization process of aniline and *o*-anisidine monomers in a glow discharge plasma follows a free radical mechanism [8]. When the discharge is ignited between the anode and the cathode at a potential of 1000 V (the substrate holder being at floating potential), the monomer vapors do not drop directly onto the substrate as they enter into the plasma and are involved in collisions with the plasma electrons. As a result of this processes, the chemical reactive species (radicals) initiate the fragmentation stage of the plasma polymerization reaction. The aniline and the *o*-anisidine fragmentation takes place via monoradical (by detaching from a hydrogen atom either by forming/detaching of an amino group on/from the aromatic ring) and biradical (by the opening of the aromatic ring and π-bond scission) species formation, thus initiating the propagation stage of the polymerization process. These radicals deposited on the substrate promote the classical chain polymerization process. In the final stage of the polymerization process, the termination, there is a simultaneous destruction of two radicals by their coupling, ending the reaction. Usually in this process, the hydrogen is transferred from one polymer radical to another killing the active end, during the discharge [8].

As the ions temperature into the plasma does not exceed 300 K, the plasma heating of the deposited layers on the substrate is avoided [7].

### 2.3. Structural, Morphological, and Electrical Characterization of Polyaniline and Poly(o-Anisidine) Layers

The morphological features of the polyaniline and poly(*o*-anisidine) layers, namely the growing process evolution of the polymers’ function of the experimental deposition parameters, were analyzed with an SPM-NTegra Prima AFM (NT-MDT) atomic force microscope (AFM, NT-MDT, Moscow, Russia), which operates in semi-contact mode, using a NSG 01 cantilever (resonance frequency: 87–230 kHz, force constant: 1.45–15.1 N/m).

The topology the polyaniline and poly(*o*-anisidine) coatings deposited on Si substrates have been investigated by scanning electron microscopy (SEM) using a FEI Inspect S scanning electron microscope ( Hillsboro, Oregon, OR, USA) in both high- and low-vacuum modes.

The IR spectra of the polyaniline and poly(*o*-anisidine) layers obtained on Si substrate were acquired in the spectral range of 4000–400 cm^−1^ using a SP100 IR Perkin Elmer spectrometer (Waltham, MA, USA) equipped with an attenuated total reflection (ATR) accessory.

The structure of the PANI layers was investigated by means of a X-ray computerized diffractometer having a X-ray source of Cu-Kα on a 0.154 nm in a Bragg-Bretano configuration. The diffractograms were performed in a 5°–70° angle range with an accuracy of 0.020 for 36 h.

The method used for determining the electrical conductivities of polyaniline and poly(*o*-anisidine) layers was previously reported in [9]. The measurements were performed by means of a Keithley 2400 source meter controlled by LabView software (custom made software). The dependence of the polymer conductivities on temperature was also established. The temperature control was granted by an INSTEC mK1000 high-precision Peltier stage and controller (Boulder, Colorado, CO, USA).

The solubility of the polymers was analyzed using the following organic solvents acquired from Sigma Aldrich Chemistry: methanol, ethyl alcohol, chloroform, acetone, and distilled water.

## 3. Results

### 3.1. AFM Analysis

Atomic force microscopy allowed the investigation of the morphological surface features of PANI and POA layers deposited on Si optically-polished surfaces, as a function of the experimental deposition conditions presented above in Table 1. The polymeric layers have been obtained at a pressure of about 5 × 10^−2^ torr inside the vacuum chamber.

**Figure 2:**
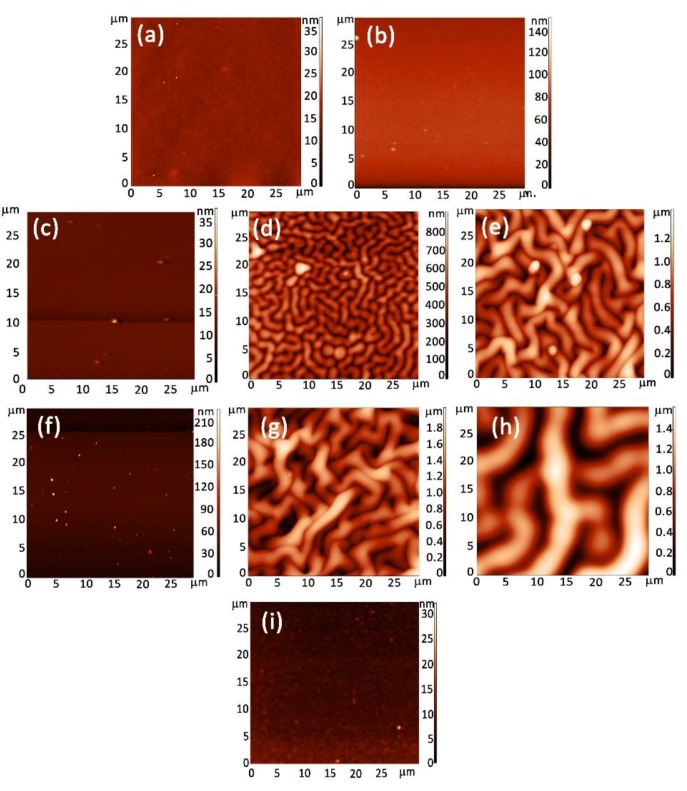
2D images of (**a**) PANI 1; (**b**) PANI 2; (**c**) PANI 3; (**d**) PANI 4; (**e**) PANI 5; (**f**) PANI 6; (**g**) PANI 7; (**h**) PANI 8; and (**i**) POA polymeric layers.

**Figure 3:**
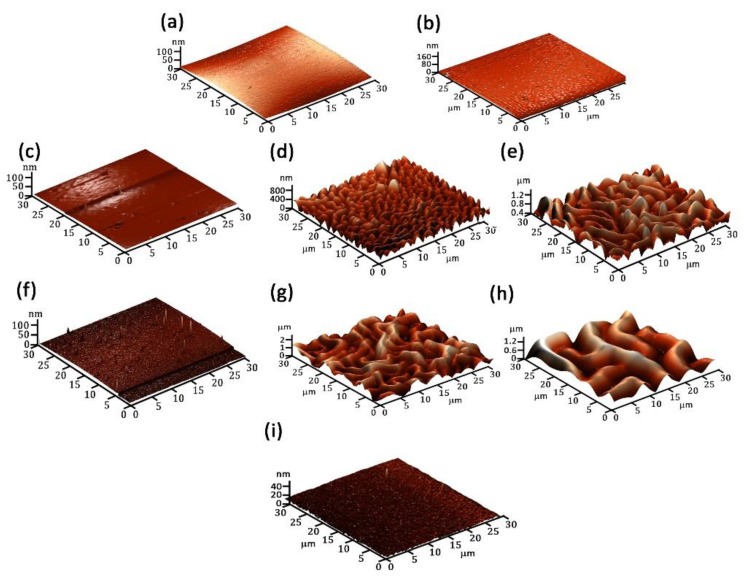
3D images of: (**a**) PANI 1; (**b**) PANI 2; (**c**) PANI 3; (**d**) PANI 4; (**e**) PANI 5; (**f**) PANI 6; (**g**) PANI 7; (**h**) PANI 8; and (**i**) POA polymeric layers.

In Figure 2 and Figure 3 are presented the 2D and 3D images of the PANI and POA layers. The PANI layers form worm-like interlinked structures that change their characteristics as a function of the experimental deposition conditions (Table 1). In Figure 2a,b it can be observed that, for PANI 1 and PANI 2 layers, an increase in the substrate inclination angle to the anode does not affect the surface morphology. By increasing the monomer injection temperature up to 50 °C and the substrate inclination angle to the anode up to 90°, (Figure 2c–e), it can be observed that worm like interlinked structures begin to form. The maximum dimension of a worm-like structure is about 1.8 μm for a 90° inclination angle.

As the experimental conditions for the polymeric layers pattering were established, the role of the anode substrate distance was investigated.

The role of the anode substrate distance on the polymeric layers networking, (Figure 2f–h) was studied and it was observed that by decreasing from 10 to 5 cm, for an inclination angle of 90° and 50 °C monomer injection temperature, the dimension of the worm-like structures attain a value of about 5 μm.

The analysis of the AFM images of the poly(*o*-anisidine) layers indicate that no kind of structures form on their surfaces even when the substrate inclination angle to the anode and the monomer injection temperature were varied. In Figure 2i an AFM image of a POA layer is presented, obtained for the experimental conditions mentioned in Table 1.

Recently [10], it was shown that polyaniline synthesized in emeraldine salt formed in strong acidic medium present a wrinkled structures on their surfaces. These structures are similar with those presented in Figure 2.

Thus, we assume that the DC glow discharge ignited in the vapors of the aniline monomers can act as a strong oxidizing medium for the polymerization of aniline.

In the polymerization process of aniline, the oxidation of monomers takes place due to oxidizing agents and, consecutively, the generated aniline cation radicals initiate the process. The nitrogen atoms usually act as oxidation centers [11].

In comparison, the free electrons of the DC glow discharge which interact with the monomer vapors produce fragmentation of the monomer. The most probable reactions inside the plasma are the detaching of the hydrogen atoms by fragmentation of N–H/C–H molecules and oxidation of Nitrogen atoms. These fragments consisting in cations, anions, and reactive species that reach the substrate holder can initiate the chain polymerization process. The recombination of the cation radicals or the electrophilic substitution (oxidizing nitrogen-containing structure attacks the phenyl ring of an aniline molecule and substitutes one proton of the ring) can promote the polymeric chain growth. During the chain growth polymerization, the monomer units are continuously added to the polymer chain bearing active end groups. The chain growth implies a continuous oxidation/reduction of the polymer chain during the glow discharge.

The increase of the monomer injection temperature up to 50 °C, of the holder inclination angle to the anode up to 90° and the decrease of the anode-holder distance to 5 cm (Table 1) support the generation of an increased amount of oxidizing agents on the substrate holder which enhanced polymer chain growth during the DC plasma. In this way, the patterning of the polymer layer surfaces is slightly changed, the dimension of the worm-like structure increasing (Figure 2). Thus, it can be explained that the formation and the evolution of the worm-like interlinked structure of the PANI layers as a function of deposition plasma conditions.

In the same time, it must be consider that as the substrate holder is at a floating potential, its superficial electrostatic charging and, consecutively, the charge distribution on the polymer growing layer surface during the deposition process assist the worm-like interlinked structure formation and evolution.

Previously, the formation of similar structures due to the building of a charge density on the free surface of a polymeric layer in the initial phase of the polymerization process was reported [12,13].

The roughnesses of the PANI and POA layers were calculated from the 2D AFM images presented in Figure 2 using image processing software. The results are shown in Table 2.

### 3.2. SEM Analysis

The topology of generated PANI and POA layers on Si substrates with optically-polished surfaces in a DC glow discharge plasma using an oblique angle substrate configuration, in the experimental deposition conditions presented in Table 1, were analyzed by scanning electron microscopy.

**Figure 4:**
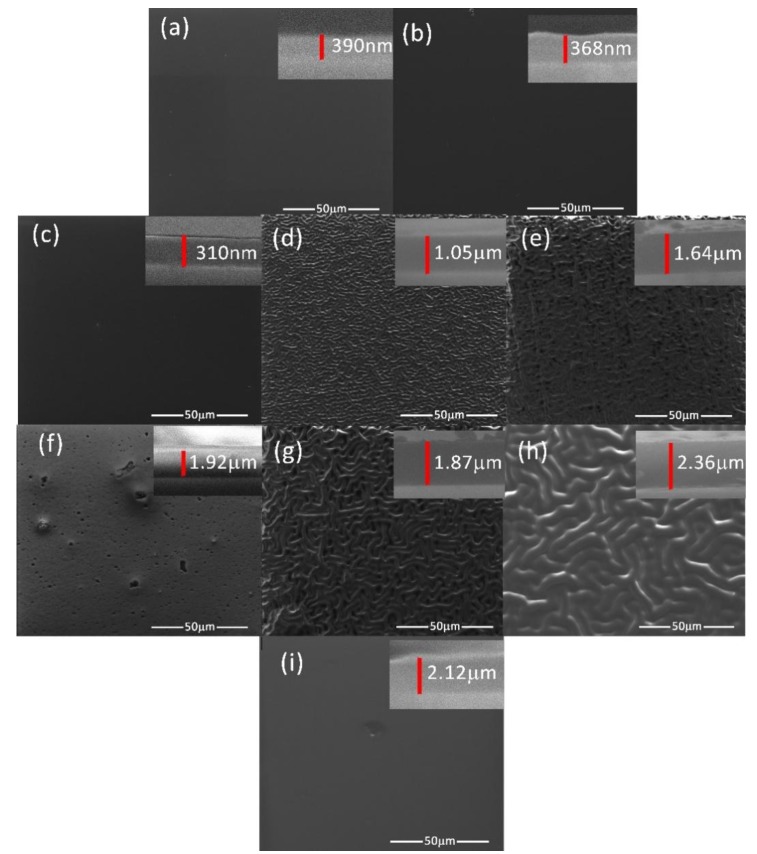
SEM images of: (**a**) PANI 1; (**b**) PANI 2; (**c**) PANI 3; (**d**) PANI 4; (**e**) PANI 5; (**f**) PANI 6; (**g**) PANI 7; (**h**) PANI 8; (**i**) POA layers.

Figure 4a–h presents the SEM images of the PANI polymeric layers and the worm-like structures’ pattering evolution function of the experimental conditions presented in Table 1. The layers do not present any cracking on their surfaces.

In Figure 4i it can be observed that the POA layer generated in experimental conditions similar to those used for synthesis of the PANI 8 layer do not present any worm-like interlinked structures on its surface.

The layer thicknesses were established by analyzing with the SEM technique, the transversal cross section of each polymer (the right corner inset image from Figure 4). The polymers are compact in their bulks, the worm-like interlinked structures being formed only on the layer surfaces. The sample PANI 1 and PANI 2 generated for a monomer temperature of 20 °C and inclination angles of 0° and 45° (Table 1) have thicknesses of about 390 and 368 nm and flat surfaces. The increase in the monomer temperature up to 50 °C for an inclination angle of 0° do not affect the structuring of the PANI 3 sample surface, it thickness being of 310 nm. There are no structures on the surface of PANI 6 sample obtained in the deposition condition presented in Table 1. The increase of the thickness can be due to the increase of the current intensity and to the decrease of the anode-substrate distance.

The deposition conditions (Table 1) used for the generation of samples PANI 4, 5, 7, and 8 are proper for the pattering of the polymer surfaces, which can be associated to the increase of the layer thicknesses. The thickness of the sample PANI 5 is higher than the one corresponding to PANI 4 (Figure 4d,e) as the inclination angle of the substrate holder to the anode has been increased from 45° to 90°. The AFM pictures in Figure 3d,e indicate also the increasing of the dimension of the worm-like structure with the inclination angle.

In comparison with the deposition conditions of PANI 5 sample (Figure 4e), maintaining the deposition time of 10 min, the inclination angle of the substrate holder to the anode of 90°, and decreasing the anode substrate distance to 5 cm, PANI 7 sample was obtained, with the surface features presented in Figure 4g. The thickness of the polymeric layer (PANI 8) has further increased up to 2.36 μm (Figure 4h) as the current intensity was set to 30 mA.

For all the PANI samples, the differences in their pattering were also analyzed by AFM measurements.

**Figure 5:**
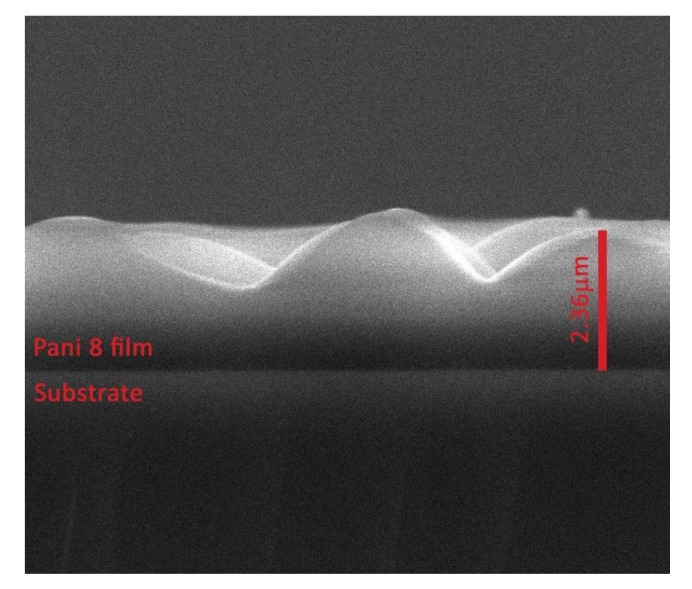
SEM images of the transversal cross-section of the PANI 8 sample.

A high-resolution SEM image of the transversal cross-section of the PANI 8 sample is presented in Figure 5. This is an indication of a surface effect that appear during the growing process of the polyaniline layer on Si substrate in a DC glow discharge plasma produced in the absence of a buffer gas, in the vapors of the monomers, using an oblique angle-positioned electrode configuration. Previously [14,15], worm-like interlinked structures in plasma polymers volume due to the buckling effect have been reported.

### 3.3. FTIR Analysis

The structural analysis of the two types of conducting polymers produced by plasma polymerization processes was performed by infrared spectroscopy.

**Figure 6:**
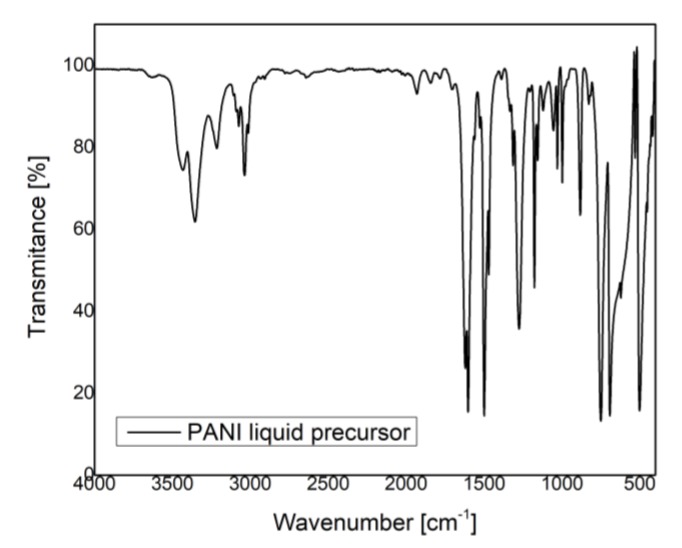
FTIR spectra of PANI liquid precursor.

**Figure 7:**
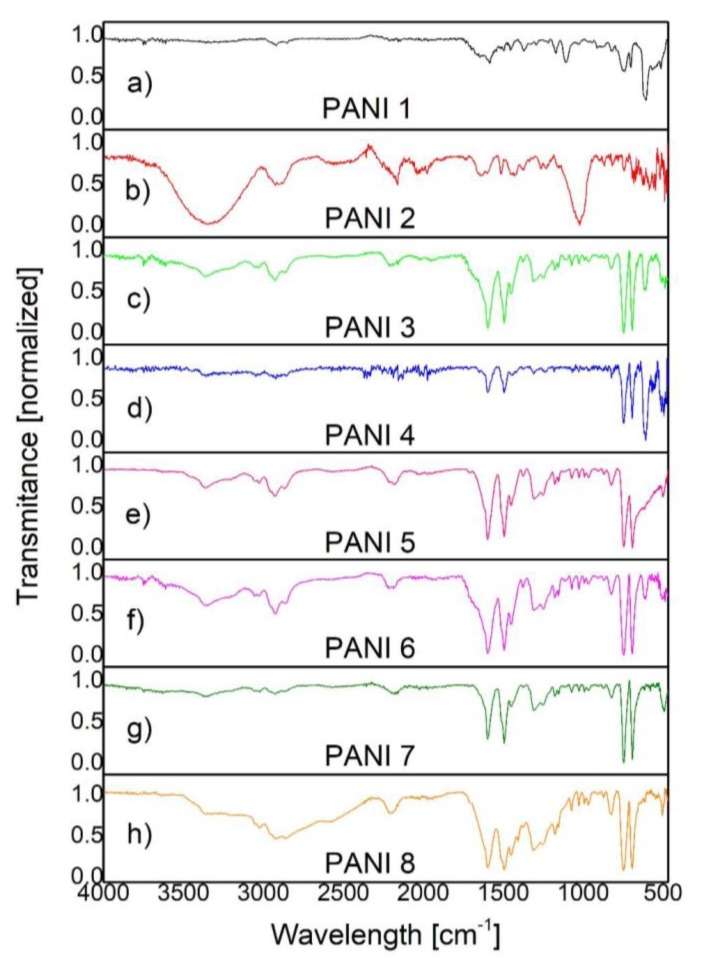
FTIR spectra of: (**a**) PANI 1; (**b**) PANI 2; (**c**) PANI 3; (**d**) PANI 4; (**e**) PANI 5; (**f**) PANI 6; (**g**) PANI 7; and (**h**) PANI 8.

In Figure 6 and Figure 7 are presented the IR spectra of PANI liquid precursor and PANI layers obtained by plasma polymerization.

In the case of both liquid and polyaniline layers, (Figure 6 and Figure 7), the IR bands specific to N–H bond vibrations (3370, 3200, 3023 cm^−1^), C–H vibrations (2961, 2921, 2862, 1373, 995, 971, 909 cm^−1^), C=N bond vibrations (1650 cm^−1^), C–N bond vibrations (1310, 1255 cm^−1^), and C=C bond vibrations (1597, 1515, 1496, 1450 cm^−1^) are observed [16,17,18,19,20].

By comparing the IR bands identified in the polymer spectra with those observed in the liquid precursor spectrum, one can obtain information about the molecular bond arrangements.

The IR bands assigned to N–H bond vibrations (3370, 3200, 3023 cm^−1^) and C–H vibrations (2961, 2921, 2862, 1373, 995, 971, 909 cm^−1^) are broader in the polymer spectra than in the liquid one.

As the inclination angle between the substrate and the anode is varied from 0° to 45°, (Figure 7a,b; Table 1), for a monomer injection temperature of 20 °C, in the 3400–2500 cm^−1^ range of the PANI 2 IR spectrum, it can be observed that the intensities of the vibrational bands of N–H groups are higher than those specific to C–H groups. Contrarily, as the monomer injection temperature is increased up to 50 °C, in the IR spectra of PANI 3, 4, 5, 6, 7, and 8 samples, (Figure 7c–h) the intensities of the C–H IR bands from 2961, 2921, and 2862 cm^−1^ are comparatively higher than those specific to N–H IR bands from 3370, 3200, and 3023 cm^−1^. Thus, it is possible that the number of N–H broken bonds inside the plasma can be enhanced with the increase of the monomer injection temperature. In the same time, the ratio between the intensities of C–H and N–H IR bands is higher as the inclination angle between the anode and the substrate holder is increased up to 90° and the anode–substrate distance is decreased to 5 cm (Figure 7f–h, Table 1).

The IR band from 1650 cm^−1^ assigned to C=N bond vibrations and IR bands characteristic to C=C bond vibrations (1597, 1515, 1496, 1450 cm^−1^) are broader in the polymer spectra than in the liquid one, for all the PANI samples. The IR bands from 1597 and 1496 cm^−1^ are characteristic to protonated polyaniline and attributed to C=C stretching vibrations of quinoid and benzoid rings.

The broadening of the IR bands observed in the polymer spectra suggests the cross-linking formation of the PANI samples as a result of their synthesis in a DC glow discharge plasma.

The intensities of the IR bands specific to vibrations of quinoid and benzoid units allow the estimation of their ratio in the polymeric layer. As their intensities are almost the same, these groups are formed in the polymer in approximately equal proportions. This could be an indication of the PANI polymerization in an emeraldine form [16]. Moreover, it is shown that the 1070 cm^−1^ band is a characteristic band for emeraldine salt, being attributed to the doping of PANI with H^+^. By this process an excitation band between the valence and conduction band can be formed, having an important role in the electrical conduction of PANI samples. At the same time, the IR band from 1155 cm^−1^ also indicates the conducting protonated process of PANI [17]. As can be observed in IR spectra from Figure 7 the 1070 and 1155 cm^−1^ IR bands characteristic to emeraldine salt form of PANI are formed in all the analyzed samples, having the smallest intensity in the PANI 2 sample and the higher one in the PANI 8 sample. At the same time, it can be noticed that the intensity of the 1070 and 1155 cm^−1^ IR bands increase with the monomer injection temperature and anode-substrate inclination angle, attaining a maximum value identified in the PANI 8 IR spectrum, (Figure 7h). These results, related to the intensities of molecular vibrational bands of the PANI samples, can be correlated with the variation in thickness of the polymeric layers in agreement with the data presented in Section 3.2. The thickness of PANI 1 sample is about 390 nm and that of PANI 8 sample attains a value of 2.36 μm.

The IR bands observed in the PANI sample spectra in the 1200–500 cm^−1^ range are similar with those observed in the liquid precursor spectrum.

**Figure 8:**
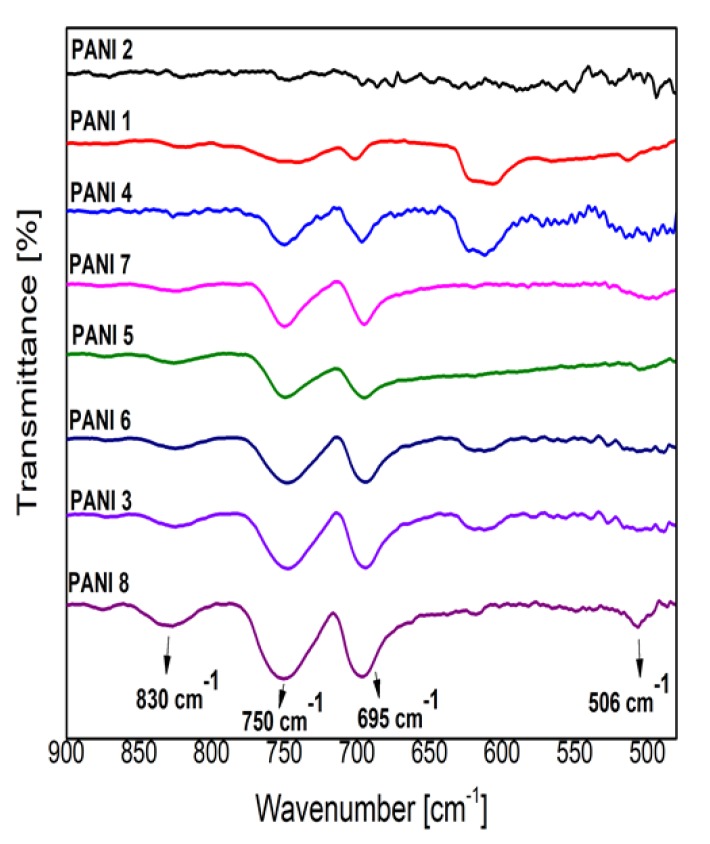
Details of PANI samples FTIR spectra in 900–450 cm^−1^ range.

The C–H out of plane bending modes in the 1000–400 cm^−1^ spectral region indicate all the substitutions present in the benzene ring as a result of the polymerization process, (Figure 8) [16,17,18,19,20]. The intensity of the IR bands observed at 747 cm^−1^ (ortho substitutions in benzene ring) and 692 cm^−1^ (meta substitutions in benzene ring) are almost equal in all the investigated samples (except the PANI 2 sample) in comparison with that from 830 cm^−1^ assigned to para substitutions. As the chain of conducting PANI contains a high proportion of para substitutions in the benzene ring [11], it results that the PANI 2 sample has the smallest conductivity, the higher conductivity being attributed to the PANI 8 sample. In Figure 7 can be observed that the intensity of the 830 cm^−1^ IR band has the highest value in the case of PANI 8 sample IR spectrum and the smallest one in the case of PANI 2 sample IR spectrum.

The dependence of the intensity of the IR band from 830 cm^−1^ on the plasma deposition conditions (Table 1) can also be observed in Figure 8. The monomer injection temperature of 50 °C (PANI 3, 5, 6, 7, 8), a substrate inclination angle of about 90° (PANI 5, 7, 8), and the anode substrate distance of 5 cm (PANI 7, 8) are the most favorable plasma deposition conditions for a high degree of aniline polymerization in para substitutions in the benzene ring.

In the polymerization process the coupling of the phenyl nuclei with respect to the amino groups is mainly performed in meta and ortho positions. The difference in the intensities of these bands and that from 830 cm^−1^ can also be an indication of a branched structure of the polymers [18,19,20].

The IR spectra of polyaniline synthetized the DC glow discharge plasma, (Figure 7 and Figure 8), showed the influence of the deposition parameters on the polymers structure. The spectra of PANI 1, 2 samples are distinct in terms of band intensities, especially in the 3700–2500 cm^−1^ range, as a result of the variation of the inclination angle between the anode and the holder substrate for a monomer injection temperature of 20 °C. The IR spectra of the PANI 3–8 samples generated at 50 °C monomer injection temperature do not present such differences in their IR spectra even when the inclination angle between the anode and the substrate holder was increased up to 90°. As these polymeric layers are spectrally identified to be in the emeraldine salt form of polyaniline (Figure 7), it seems that the inclination angle between the anode and the substrate holder affect mainly the proportion between para, ortho, and meta substitutions in benzene rings and, as a consequence, the conductivity of PANI samples.

**Figure 9:**
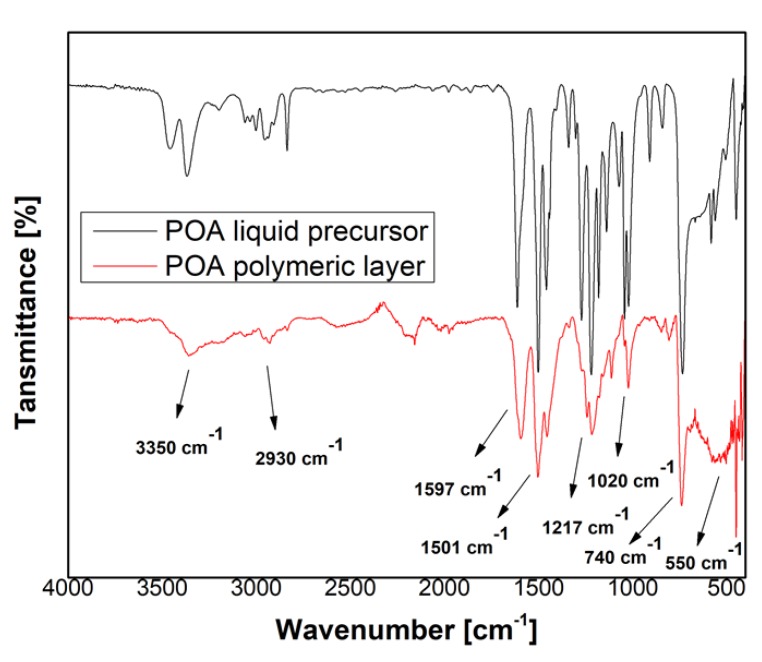
FTIR spectrum of the poly(*o*-anisidine) liquid precursor (black line) and polymer (red line).

Figure 9 presents the spectra of poly(*o*-anisidine) liquid precursor (black line) and POA layers (red line). The IR bands assignment identified within the spectra are shown in Table 3 and Table 4.

The FTIR spectra of poly(*o*-anisidine) liquid precursor and polymeric film obtained in the DC plasma reactor are shown in Figure 9. The corresponding IR band assignments are presented in Table 4 in agreement with the reference data [4,18,20,22]. In the polymeric layer, the main characteristic bands are attributed to: N–H stretching vibrations (3350 cm^−1^) in poly(*o*-anisidine) group; the vibrations of the quinoid (1597 cm^−1^) and benzoid (1501, 1455 cm^−1^ [18]) rings which confirm the plasma polymerization of *o*-anisidine to poly(*o*-anisidine); carboxyl groups (1270, 1240 cm^−1^) presence on benzene rings; 1,2,4 trisubstituted benzene ring (1217, 1177, 1152 cm^−1^); 1,4 substitution on the benzene rings (1117, 1020 cm^−1^); 1,2 and 1,3 substitutions on the benzene ring (848, 805, 740 cm^−1^) [4]; 1,4 disubstitution on benzene ring (550 cm^−1^) can be clearly noticed. In comparison with the IR bands characteristic to the liquid precursor it can be observed that in the POA polymer spectrum, in the spectral range of 3400–2700 cm^−1^, the IR bands characteristic to N–H stretching vibrations (3350 cm^−1^) and C–H stretching vibrations (2930, 2882 cm^−1^) are overlaid as an indication of their broadening. The C=C stretching vibrations of quinoid groups appear in the polymer spectrum at 1597 cm^−1^, not at 1612 cm^−1^ as in the liquid precursor spectrum. The C=C stretching vibration of benzoid groups has similar IR band features in both liquid and polymer spectra.

In 1320–1056 cm^−1^ and 617–470 cm^−1^ spectral ranges the IR bands are better evidenced in the precursor liquid spectrum than in the polymer one. This can be an indication that, in the polymer, the IR bands are broader and overlaid. The broadening of the IR bands is an indication of the cross-linking.

In comparison to the IR spectrum of PANI, the IR spectrum of poly(*o*-anisidine) from Figure 9 shows that the IR band specific to benzoid group is more intense than the one of quinoid group, indicating that their proportions are not equal.

### 3.4. X-Ray Diffraction Analysis

**Figure 10:**
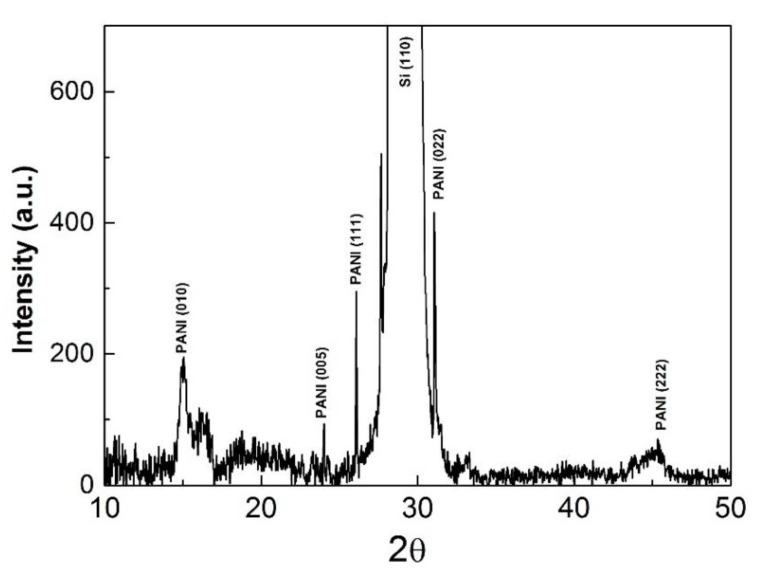
XRD diffraction patterns of the PANI 8 sample.

The crystalline structure of the polymers was investigated for all the samples produced in the experimental conditions presented in Table 1 by means of an X-ray diffraction (XRD) setup. The XRD patterns of the PANI 8 sample are presented in Figure 10.

The main reflections correspond to a pseudo orthorhombic phase of PANI. Crystallinity of PANI samples could account for superior conductive properties of the thin polymer layers. Thus, a highly-ordered structure, similar to metals, will most likely have enhanced conductivity. Our results present a textured polymer film made up of two components from the crystalline point of view. The first one formed from (010), (200) with broad appearance and low intensity due to the reduced crystalline dimension with a low degree of organization, and the other one composed of intense and narrow reflections corresponding to a highly-ordered structure. Additionally, in the latter case, the crystallite size increased with a factor of ~10. These results indicate that the polymeric layers are formed by crystalline domains embedded in an amorphous matrix.

Defects in the crystal symmetry are most likely caused by lattice strain which represents a measure of both defects and dislocation. This, in turn, leads to a lattice deformation that causes the residual stress in the crystalline matrix. Moreover, the lattice strain induces an effect of line broadening in the XRD spectra.

Assuming that the crystallite size and lattice strain have an independent contribution to line broadening, we calculated the strain induced in the PANI crystalline structure with the following relation [23]:

β = 4εtanθ(1)

where ε is the lattice strain induced in crystalline particles and β the full width at half maximum value.

The crystalline plane and the lattice strain associated to the diffraction peaks identified in Figure 10 are summarized in Table 5.

The crystalline domain size was evaluated using the Scherrer formula [16]:

d = 0.89λ/βcosθ(2)

where d represents the crystallite dimension, λ the specific X-ray wavelength.

The interchain separation length was determined from Equation (2) for the highest intensity crystalline orientation. This parameter can give a measure of the polymeric layers conductivity and it represents the hopping distance of electrons from one chain to another. Thus, the probability for a polymer layer to be conductive increases as the distance between the chains decreases [16]. In our case, the interchain separation length value was 3.59 Å, which is in good agreement with the literature for the conductive polyaniline [16].

(3) $$R=\frac{5\lambda }{8\sin\theta }$$

**Figure 11:**
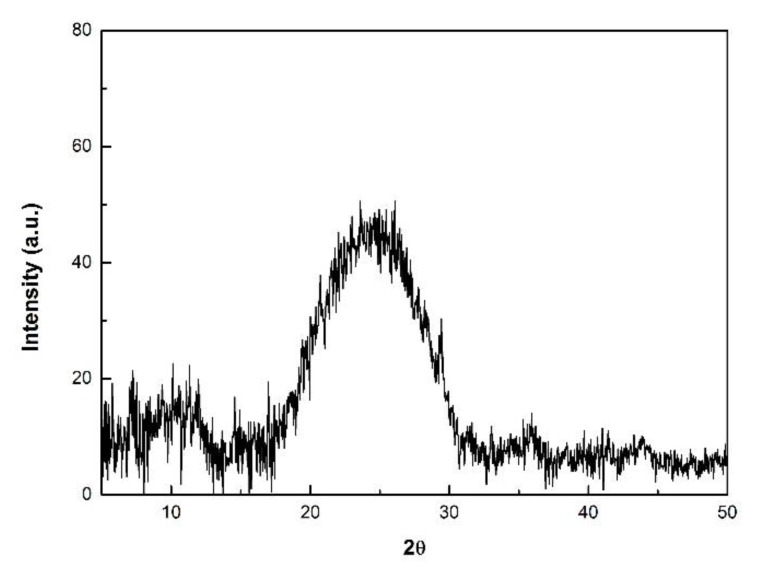
XRD diffraction patterns of the POA sample.

As presented in Figure 11 the diffraction pattern of the POA sample exhibits a medium broad peak at 2θ = 12.5°, and a broad intense peak with its maximum centered at 25°. These peaks are attributed to the semi-crystalline nature of the polymer [24].

In the case of PANI and POA samples obtained for a substrate inclination angle of 0 degree, namely, PANI 1, PANI 3, and PANI 6, there were no observed diffraction patterns.

### 3.5. Solubility Analysis

The solubility behavior of plasma polymeric films can give useful knowledge about their degree of cross-linking. For example, a well cross-linked polymer is typically insoluble in organic solvents [24].

The solubility of the PANI and POA thin films was investigated using four organic solvents, namely: methanol, ethyl alcohol, chloroform, acetone, and distilled water. It was analyzed qualitatively by recording the FT-IR spectra of the layers before and after their submerging in organic solvents for 5 min. By comparing these spectra for each polymer, we observed that the intensities of the IR bands, specific to poly(aniline) and to poly(*o*-anisidine) kept in the organic solvents, become partially (50%), completely (100%), or not diminished. In terms of solubillity we interpret these results as partially soluble, soluble, or insoluble samples. The results are shown in the Table 6.

### 3.6. Electrical Conductivity Measurements

Polyaniline polymers synthesized by chemical, electrochemical or plasma methods, can have electrical conductivities ranging between 10^−10^ and 10 S/cm. The electrical conductivity of PANI depend on it oxidation state (pernigraniline, emeraldine or leucoemeraldine) and degree of protonation. The undoped form of polyaniline usually has an electrical conductivity in the ~10^−9^ S/cm range while the conductivity of the H^+^ doped polyaniline varies from ~10^−5^ S/cm to 10 S/cm [9,11,16,22].

The electrical conductivities were measured for all the samples produced in the experimental condition presented in Table 1. At room temperature, the conductivity of PANI 2 sample is about 3.8 × 10^−5^ S/cm and the conductivity of PANI 8 sample is 5 × 10^−5^ S/cm. There are smaller differences in the conductivities of the other PANI samples. In principle, the sequence in the increase in their conductivities follow the behavior of the intensity of the 830 cm^−1^ IR band (attributed to para substitutions in benzene ring) as it was observed in the FTIR spectra from Figure 8. On the other hand, the intensities of IR bands from 1070 and 1155 cm^−1^ could indicate the differences in the concentration of emeraldine salt formed in PANI samples. The spectra from Figure 7 indicates that the PANI 2 sample has the smallest concentration of emeraldine salt, while the PANI 8 sample has the highest one.

**Figure 12:**
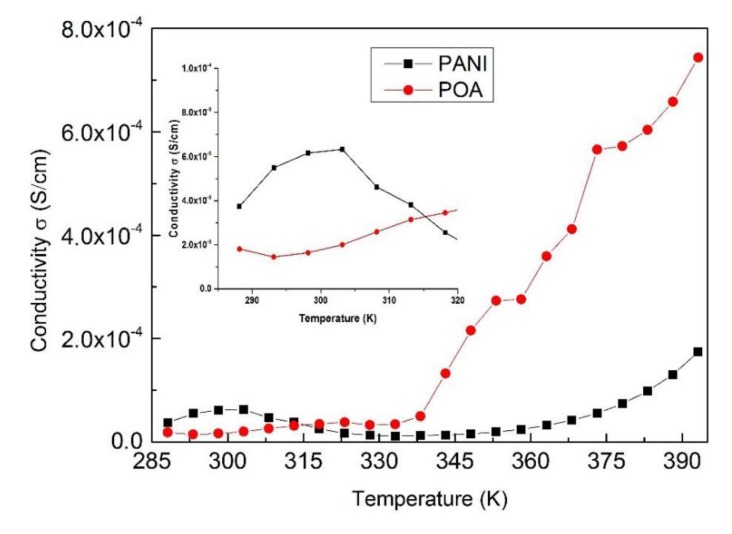
Dependence of PANI 8 and POA samples’ electrical conductivity on temperature.

The dependence of the electrical conductivity of polyaniline (PANI 8 sample) and poly(*o*-anisidine) samples on temperature is presented in Figure 12. The conductivities of all the PANI samples have similar dependences on temperature.

The poly(*o*-anisidine) sample shows an abrupt increase of the conductivity with temperature, reaching a value of 7 × 10^−4^ S/cm at 395 K, in comparison with that of the polyaniline (PANI 8 sample) that only increases up to 2 × 10^−4^ S/cm, Figure 12.

## 4. Conclusions

In this study we investigated from physicochemical and morphological points of view of the polyaniline and poly(*o*-anisidine) polymer layers generated in a direct current glow discharge plasma in the absence of a buffer gas, in the vapors of the monomers, using an oblique angle positioned substrate configuration. In comparison with the poly(*o*-anisidine) layer surfaces which are flat, on the surface of the polyaniline films we identified the formation of worm-like interlinked structures having dimensions and a spatial distribution dependent on the plasma polymerization experimental conditions. For the 0 degree inclination angle between the anode and the substrate holder, there was no observed structure on the polymer surfaces of any kind.

The main advantage of this plasma polymerization technique is the ease of synthesis (10 min) and the possibility of different patterning of the polyaniline polymer surfaces only by varying the deposition conditions, namely the inclination angle of the substrate holder to the anode and the monomer injection temperature. This does not imply any supplementary chemical or physical treatments which can affect the layer surface morphologies, mainly by cracking.

The analysis of the physical and chemical properties of the polymeric layers has been performed by infrared and X-ray diffraction spectroscopy.

The FTIR spectral analysis of polyaniline layers indicates, besides the main IR bands, the conductive character of polyaniline. The intensities of the IR bands specific to conducting protonated PANI samples and ascertain it emeraldine salt form (1070, 1155 cm^−1^) increase with the thicknesses of the layers. At the same time, the intensity of the molecular band from 830 cm^−1^, characteristic to the para substitutions in the benzene ring, can give information about the conductivities of the layers.

The FTIR analyses are in agreement with the conductivity measurements which show that the PANI 8 sample has the highest conductivity (5 × 10^−5^ S/cm) and the PANI 2 sample has the lowest one (3.8 × 10^−5^ S/cm). This results in the conductivity of the investigated samples increasing as the concentration of emeraldine salt in the polymers increases.

The electrical conductivity of polyaniline is higher than of poly(*o*-anisidine) in a 285–315 °C temperature range. In contrast, the conductivity of poly(*o*-anisidine) layers increase to higher values than that of polyaniline in the 315–395 °C temperature range.

Another advantage of this plasma polymerization technique was identified by the XRD spectra analysis that show the formation of crystalline domains embedded in an amorphous matrix in the PANI 8 sample. The X-ray diffraction pattern of poly(*o*-anisidine) highlighted the semi-crystalline nature of the layers. The polymers obtained for a 0 degree inclination angle between the anode and the substrate holder are amorphous. Usually, the polyaniline layers are obtained by different plasma deposition techniques, and the chemical or electrochemical methods are amorphous. Polymers with crystalline or semi-crystalline structure can be obtained only by physical or chemical treatments of the layers using supplementary methods.

## Figures and Tables

**Table 1:** Experimental deposition conditions for the polymeric films.

Sample No.	Voltage (V)	Current Intensity (mA)	Anode-Substrate Distance (cm)	Substrate Inclination Angle	Monomer Temperature (°C)	Time (min)
PANI 1	1000	20	10	0°	20	10
PANI 2	1000	20	10	45°	20	10
PANI 3	1000	20	10	0°	50	10
PANI 4	1000	20	10	45°	50	10
PANI 5	1000	20	10	90°	50	10
PANI 6	1000	30	5	0°	50	10
PANI 7	1000	20	5	90°	50	10
PANI 8	1200	30	5	90°	50	10
POA	1200	30	5	90°	50	10

**Table 2:** Roughness measurements of PANI and POA layers.

Sample	PANI 1	PANI 2	PANI 3	PANI 4	PANI 5	PANI 6	PANI 7	PANI 8	POA
Peak to peak (nm)	2.484	8.532	6.48	382.96	996.3	14.04	968.436	1217.38	8.1
Average (nm)	0.393	21.492	0.956	85.04	167.92	2.49	156.724	247.65	1.85

**Table 3:** IR spectral bands identified in the PANI spectra.

Wavenumber (cm^−1^)	IR Vibrational Unit
3370, 3200, 3023	N–H stretching vibrations [16,18]
2961, 2921	C–H stretching vibrations in CH_3_ [16]
2862	C–H vibrations in CH_2_ [16]
1650	C=N stretching vibrations of quinoid ring [16]
1597	C=C stretching vibrations of quinoid ring [5,16,19]
1515, 1496, 1450	C=C stretching vibrations of benzoid ring [1,5,16]
1405	C–N^+^ stretching vibrations [16]
1373	C–H symmetric deformation vibrations in –CH_3_ [19]
1310	C–N stretching vibrations of aromatic ring [19]
1255	C–N stretching vibrations in aromatic primary amine [16,20]
1173, 1109, 1026	In-plane bending vibrations of aromatic C–H [19]
1155	C–N stretching vibrations in benzoid ring [17]
1070	Quinoid ring –NH^+^– benzoid ring stretching vibrations [16]
995, 971, 909	C–H out of plane bending vibrations [18]
873, 692	meta substitutions, 1,3 disubstitution in benzene ring [18]
830, 554	para substitutions, 1,4 disubstitution in benzene ring [2,18]
747, 506	ortho substitutions, 1,2 disubstitution in benzene ring [16,19]
613	vibrations in the aryl nitro compounds [5]

**Table 4:** IR spectral bands assignments identified in poly(*o*-anisidine) spectrum.

Wavenumber cm^−1^	IR Vibrational Unit
3350	N–H stretching vibrations [18,21]
2930, 2882	C–H stretching vibrations in CH_3_ [20]
1597	C=C stretching vibrations of quinoid groups [18,21]
1501, 1455	C=C stretching vibration of benzoid groups [4,18,21]
1335	N–H group vibration [22]
1270, 1240	Carboxyl groups vibrations on benzene ring [4,22]
1217, 1177, 1152	1,2,4 trisubstituted benzene ring [21]
1117, 1020	1,4 substitution on the benzene ring [4]
848, 805, 740	1,2 and 1,3-substitutions on benzene ring [4]
550	1,4 disubstitution on benzene ring [2,20]

**Table 5:** Crystallinity proprieties of the PANI 8 sample.

hkl	Diffraction Peak (2θ)	d (nm)	Lattice Strain
PANI (010)	15.03	15.21	0.0182
PANI (005)	23.99	99.07	0.0018
PANI (111)	26.07	109.77	0.0015
PANI (022)	89.51	31.07	0.0015
PANI (200)	40.81	3.95	0.0238

**Table 6:** Solubility of PANI and POA samples.

Sample	Organic Solvent				
	CH_3_OH	C_2_H_6_O	CHCl_3_	C_3_H_6_O	H_2_O
PANI 1	p	p	p	s	i
PANI 2	p	p	p	s	i
PANI 3	p	p	p	s	i
PANI 4	p	p	p	s	i
PANI 5	p	p	p	s	i
PANI 6	p	p	p	s	i
PANI 7	p	p	p	s	i
PANI 8	p	p	p	s	i
POA	s	s	s	s	i

Where s—soluble, p—partially soluble and i—insoluble.

## References

[1] Lakshmi G.B.V.S., Dhillon A., Siddiqui A.M., Zulfequar M., Avasthi D.K. (2009). RF-plasma polymerization and characterization of polyaniline. Eur. Polym. J..

[2] Cruz G.J., Morales J., Castillo-Ortega M.M., Olayo R. (1997). Synthesis of polyaniline films by plasma polymerization. Synth. Met..

[3] Ameen S., Akhtar M.S., Song M., Shin H.S. (2013). Metal Oxide Nanomaterials, Conducting Polymers and Their Nanocomposites for Solar Energy. Solar Cells-Research and Application Perspectives.

[4] Chaudhari S., Sainkar S.R., Patil P.P. (2007). Anticorrosive properties of electro synthesized poly(*o*-anisidine) coatings on copper from aqueous salicylate medium. J. Phys. D Appl. Phys..

[5] Gong X., Dai L., Mau A.W., Griesser H.J. (1998). Plasma-Polymerized Polyaniline Films: Synthesis and Characterization. J. Polym. Sci. A.

[6] Staicu D., Butoi B., Armeanu C., Barna E.S. (2016). Influence of the key deposition control parameters on the structure of thin films in a direct current cold plasma reactor for photonics applications. Dig. J. Nanomater. Biostruct..

[7] Butoi B., Berezovski C., Staicu D., Berezovski R., Marin A.M., Barna E.S. (2014). Direct Current Plasma Polymerization Reactor for Thin Duromer Film Deposition. J. Optoelectron. Adv. Mater..

[8] Jatratkar A.A., Yadav J.B., Deshmukh R.R., Barshilia H.C., Puri V., Puri R.K. (2016). Impact of low-pressure glow-discharge pulsed plasma polymerization on properties of polyaniline thin films. Phys. Scr..

[9] Shi G., Rouabhia M., Wang Z., Dao L.H., Zhang Z. (2004). A novel electrically conductive and biodegradable composite made of polypyrrole nanoparticles and polylactide. Biomaterials.

[10] Xie J., Zong C., Han X., Ji H., Wang J., Yang X., Lu C. (2016). Redox-Switchable Surface Wrinkling on Polyaniline Film. Macromol. Rapid Commun..

[11] Sapurina I.Y., Shishov M.A. (2012). Oxidative Polymerization of Aniline: Molecular Synthesis of Polyaniline and the Formation of Supramolecular Structures. New Polymers for Special Applications.

[12] Groza A., Surmeian A., Diplasu C., Luculescu C., Chapon P., Tempez A., Ganciu M. (2012). Physico-chemical processes occurring during polymerization of liquid polydimethylsiloxane films on metal substrates under atmospheric pressure air corona discharges. Surf. Coat. Technol..

[13] Groza A., Ciobanu C.S., Popa C.L., Iconaru S.L., Chapon L., Luculescu C., Ganciu M., Predoi D. (2016). Structural properties and antifungal activity against Candida albicans biofilm of different composite layers based on Ag/Zn doped hydroxyapatite-polydimethylsiloxanes. Polymers.

[14] Wang X., Grundmeier G. (2006). Morphology and Patterning Processes of Thin Organosilicon and Perfluorinated Bi-Layer Plasma Polymer Films. Plasma Process. Polym..

[15] Tsai T.C., Staack D. (2011). Low-Temperature Polymer Deposition in Ambient Air Using a Floating-electrode Dielectric Barrier Discharge Jet. Plasma Process. Polym..

[16] Du X., Xu Y., Xiong L., Bai Y., Zhu J., Mao S. (2014). Polyaniline with high crystallinity degree: Synthesis, structure, and electrochemical properties. J. Appl. Polym. Sci..

[17] Jarad A.N., Ibrahim K., Ahmed N.M. (2016). Synthesis and characterization thin films of conductive polymer (PANI) for optoelectronic device application. AIP Conf. Proc..

[18] Coates J., Meyers R.A. (2000). Interpretation of Infrared Spectra, A Practical Approach. Encyclopedia of Analytical Chemistry.

[19] Tamirisa P.A., Liddell K.C., Pedrow P.D., Osman M.A. (2004). Pulsed-Plasma-Polymerized Aniline Thin Films. J. Appl. Polym. Sci..

[20] Ohsaka T., Ohnuki Y., Oyama N., Katagiri G., Kamisako K. (1984). IR absorbtion spectroscopic identification of electroactive and electroinactive polyaniline films prepared by the electrochemical polymerization of aniline. J. Electroanal. Chem. Interfacial Electrochem..

[21] Nabid M.R., Zamiraei Z., Sedghi R. (2010). Water-soluble Aniline/*o*-Anisidine Copolymer: Enzymatic Synthesis and Characterization. Iran. Polym. J..

[22] Shah K., Iroh J. (2004). Poly(*o*-anisidine) Coatings Electrodeposited onto AL-2024: Synthesis, Characterization, and Corrosion Protection Evaluation. Adv. Polym. Technol..

[23] Mote V.D., Purushotham Y., Dole B.N. (2012). Williamson–Hall analysis in estimation of lattice strain in nanometer-sized ZnO particles. J. Theor. Appl. Phys..

[24] Özdemir C., Kaplan Can H., Colak N., Güner A. (2006). Synthesis, Characterization, and Comparison of Self-Doped, Doped, and Undoped Forms of Polyaniline, Poly(*o*-anisidine), and Poly[aniline-*co*-(*o*-anisidine)]. J. Appl. Polym. Sci..

